# Over-Reporting in Handwashing Self-Reports: Potential Explanatory Factors and Alternative Measurements

**DOI:** 10.1371/journal.pone.0136445

**Published:** 2015-08-24

**Authors:** Nadja Contzen, Sandra De Pasquale, Hans-Joachim Mosler

**Affiliations:** 1 Environmental and Health Psychology, Department of Environmental Social Sciences, Eawag: Swiss Federal Institute of Aquatic Science and Technology, Duebendorf, Switzerland; 2 Department of Psychology, University of Zurich, Zurich, Switzerland; University of Calgary, CANADA

## Abstract

Handwashing interventions are a priority in development and emergency aid programs. Evaluation of these interventions is essential to assess the effectiveness of programs; however, measuring handwashing is quite difficult. Although observations are considered valid, they are time-consuming and cost-ineffective; self-reports are highly efficient but considered invalid because desirable behaviour tends to be over-reported. Socially desirable responding has been claimed to be the main cause of inflated self-reports, but its underlying factors and mechanisms are understudied. The present study investigated socially desirable responding and additional potential explanatory factors for over-reported handwashing to identify indications for measures which mitigate over-reporting. Additionally, a script-based covert recall, an alternative interview question intended to mitigate recall errors and socially desirable responding, was developed and tested. Cross-sectional data collection was conducted in the Borena Zone, Ethiopia, through 2.5-hour observations and 1-hour interviews with the primary caregivers in households. A total sample of *N* = 554 was surveyed. Data were analysed with correlation and multiple regression analyses and dependent *t*-tests. Over-reporting of handwashing was associated with factors assumed to be involved in (1) socially desirable responding, (2) encoding and recall of information, and (3) dissonance processes. The latter two factor groups explained over-reported handwashing beyond socially desirable responding. The alternative interview question—script-based covert recall—reduced over-reporting compared to conventional self-reports. Although the difficulties involved in measuring handwashing by self-reports and observations are widely known, the present study is the first to investigate the factors which explain over-reporting of handwashing. This research contributes to the limited evidence base on a highly important subject: how to evaluate handwashing interventions efficiently and accurately.

## Introduction

### Background

Diarrheal diseases and acute respiratory infections are the main causes of death among children younger than 5 years old throughout the world [1] and are the most common causes of mortality in emergencies and disasters [2]. Developing countries experience the highest death tolls from these diseases and infections [1, 3]. Regular handwashing with soap (in the present study, handwashing refers to handwashing with soap), especially by the primary caregiver in a household, effectively prevents both diseases [4, 5]. Hand hygiene is also a key measure to prevent healthcare associated infections, which are a major health issue in developed and, especially, developing countries [6]. Handwashing interventions, therefore, are a program priority in development and emergency relief organisations (e.g. [7, 8]) and in healthcare settings (e.g. [9]). In both contexts, intervention evaluations are essential to design evidence-based programming [10]. Additionally, in hospital settings, compliance monitoring and feedback are seen as integral components of hand hygiene interventions [11].

However, measuring handwashing behaviour can be quite difficult [6, 12, 13]. In household and hospital settings, the gold standard in handwashing behaviour measurement is direct observation, in which a trained observer watches and records household members’ or health workers’ handwashing at key times over 3 to 24 hours [6, 12–14]. Although direct observation tends to distort behaviour towards more desirable practices (e.g. [6, 15]), it is considered the most valid measure, primarily as it is thought to be more objective than self-reports collected by interviews [16]. In addition, direct observation allows the collection of individual data (e.g. data on a specific person, instead of household or ward data) and detailed information (e.g. key times, cleansing agents) [6, 12]. However, observation is demanding for the observer and, more importantly, time-consuming and costly [11, 13].

Self-reported handwashing can be collected easily and efficiently through interviews [6, 12]. However, self-reported handwashing rates tend to be inflated when compared to observed data, meaning that good or desirable behaviour is self-reported more frequently than it is observed (e.g. [6, 16–19]). Socially desirable responding—defined as the tendency to under-report socially undesirable behaviours and to over-report socially desirable ones [20]—is thought to be the main source of bias. Consequently, in the literature to date the application of self-reporting to measure handwashing behaviour is not recommended (e.g. [11–13]).

In sum, the measurement of handwashing behaviour is a major challenge for intervention evaluations as observations are considered valid but cost-ineffective and self-reports cost-effective but invalid [6, 13]. Although socially desirable responding has been claimed to be the main cause of these inflated self-reports, only one study, to our knowledge, has tested the influence of socially desirable responding on self-reported hygiene and handwashing behaviour [21]. Furthermore, this study investigated socially desirable responding only as a personality characteristic of respondents but not as a characteristic of the topic of a questionnaire or of the context in which a questionnaire is administrated. To our knowledge, no alternative sources of inflated self-reports have been investigated. A better understanding of the causes and underlying mechanisms of over-reported handwashing might indicate measures that could mitigate over-reporting, including improved interview questions, meaning efficient *and* valid self-report measures of handwashing. Therefore, the main aim of the present study was to investigate socially desirable responding and additional potential explanatory factors for the bias between self-reported and observed handwashing behaviour. The research questions were whether socially desirable responding and other factors are associated with over-reported handwashing and whether additional factors beyond socially desirable responding explain over-reporting. In addition, we investigated whether an alternative self-report measure thought to reduce the influence of socially desirable responding and additional factors mitigates the response bias. In the following section we discuss potential sources of over-reported handwashing and deduce corresponding hypotheses.

### Potential sources of over-reported handwashing and corresponding hypotheses

A response might be biased as (1) the response was edited [22], (2) the encoded and memorized information upon which the response is based was incorrect [23] or (3) the information was recalled incorrectly (i.e. retrieved and integrated [22]). These potential sources of over-reported handwashing are discussed in the following.

### Response editing: Socially desirable responding

Socially desirable responding is one of the most commonly studied forms of response editing and response biases [22]. First, individuals’ tendency to respond in a socially desirable manner has been found to vary [24]. This pattern holds true for self-reported hygiene and handwashing behaviour [21] and general preventive health behaviour [25]. The primary mechanism thought to underlie socially desirable responding is the need to conform to social standards [20, 24]. This need becomes especially high when people feel strongly attached to their social in-group [26]. Therefore, we hypothesised the following:

H1: The higher the tendency to respond in a socially desirable manner is, the higher over-reporting of self-reported handwashing is.
H2: The higher the need for conformity is, the higher over-reporting of self-reported handwashing is.
H3: The higher the feeling of group attachment is, the higher over-reporting of self-reported handwashing is.

Second, socially desirable responding also depends on the sensitivity of a questionnaire’s topic, in other words, whether a clear social norm regarding the behaviour or attitude at hand exists [23]. What is critical is not the actual social norm but the individually perceived social norm; therefore, the perceived degree of the sensitivity of a question can differ among respondents [23, 27]. Therefore, we hypothesised the following:

H4: The stronger the perceived social norms that favour handwashing are, the higher over-reporting of self-reported handwashing is.

Third, if a question is sensitive, the level of privacy or confidentiality offered in an interview influences socially desirable responding [23, 28]. The presence of third parties, such as spouses, family members and neighbours, has been found to both increase and decrease socially desirable responding (e.g. [29]). Therefore, we formulated the following research questions:

Q1: Is the presence of a spouse during the interview associated with over-reporting of self-reported handwashing?
Q2: Is the presence of other adults during the interview associated with over-reporting of self-reported handwashing?

### Inaccurate memory formation and recall errors

Regarding inaccurate memory formation and recall errors, a first factor potentially distorting the encoded handwashing frequency is forgetting to wash hands at a certain moment. Compared to forgetting other health behaviours, forgetting to wash hands at a certain moment stands out as it might never become salient at any moment to individuals. Respondents might not realize how often they intended to but forgot to wash their hands; therefore, the encoded handwashing frequency might be biased against the actual amount and towards the intended amount. Regular daily routines facilitate and frequent task interruptions impede remembering and implementing intended behaviours [30], so we assumed that both factors affect over-reporting. These factors were also assumed to influence information recall. Frequent behaviours, such as handwashing, are often assessed by rate-based estimation strategies (e.g. I eat three times a day and, therefore, 21 times a week), which produce inaccurate estimates, namely over-estimations, when behaviour is irregular [31, 32]. Accordingly, a regular daily routine might increase the accuracy of estimations, while frequent task interruptions might lead to over-estimation and, thus, over-reporting. Therefore, we hypothesised the following:

H5: A regular daily household routine is associated with less over-reporting of self-reported handwashing than an irregular daily household routine.
H6: The more frequently tasks are interrupted, the higher over-reporting of self-reported handwashing is.

Regarding further recall errors, memory performance varies among people; some people perform better than others at a variety of memory tasks [33, 34]. It is plausible that the capacity to estimate frequencies differs with some people generally over-estimating, some being rather accurate and others generally under-estimating. Therefore, we hypothesised the following:

H7: The more a person tends to over-estimate, the higher over-reporting of self-reported handwashing is.

### Cognitive dissonance

Returning to information formation, a further factor that might increase encoding inaccuracy is cognitive dissonance, a state of psychological tension felt when one’s cognitions (e.g. knowledge or attitudes) and/or behaviour are dissonant [35]. Regarding handwashing, people might experience cognitive dissonance if they know about the preventive effects of handwashing at key times but do not always do so. Individuals attempt to dissolve cognitive dissonance by changing the behaviour or by rationalising behaviour neglects ([35]; for a recent study on rationalisation, see [36]). Assuming that the cognitive representation of behaviour is as flexible as the attitudinal or knowledge-related cognitive elements, one might alternatively reduce dissonance by biasing the perceived and, thus, the encoded frequency of the behaviour (see also [27, 37]). Therefore, people with insufficient handwashing compliance but accurate health knowledge might reduce the discomfort felt due to dissonance by 1) cognitively rationalising the neglect of handwashing or 2) increasing the perceived handwashing frequency, that is, over-reporting. Therefore, we hypothesised the following about people with imperfect handwashing compliance:

H8: The higher handwashing-related health knowledge is, the higher over-reporting of self-reported handwashing is.
H9: The higher cognitive rationalisation of neglected handwashing is, the lower over-reporting of self-reported handwashing is.
H10: Health knowledge and rationalisation interact with the highest over-reporting found for high health knowledge and low rationalisation and with the lowest over-reporting found for low health knowledge and high rationalisation.

### Script-based covert recall: A potential method to mitigate over-reported handwashing

When factors that largely explain over-reporting are found, the response bias can be addressed by improving interview questions accordingly [23]. To mitigate the recall errors of frequency reports, it has been suggested to ask respondents to reconstruct the entire process (e.g. using the latrine for defecation) leading to the behaviour of interest (e.g. handwashing), instead of directly asking about the behaviour [38, 39]. Doing so should activate the corresponding behavioural scripts stored in memory. Among respondents who regularly wash their hands before or after a specific activity, handwashing likely is an integral part of their respective script. Therefore, these respondents should mention handwashing without prompting when retelling their typical behaviour sequences. In addition, if respondents are not explicitly asked about handwashing (covert recall) and are not aware of the purpose and potential sensitivity of the content of questions, socially desirable responding might also be mitigated. Therefore, we hypothesised the following:

H11: Script-based covert recall produces less over-reporting than action-based prompted recall (i.e. a standard self-report measure).

### Summary of research questions and hypotheses

To sum up, the present study investigated (1) whether socially desirable responding, inaccuracies in encoding, recall errors and dissonance processes are associated with over-reporting of handwashing; (2) whether the latter processes explain over-reporting beyond socially desirable responding; and (3) whether an alternative self-report measure reduces over-reported handwashing. To address these research questions, a cross-sectional study that combined observational and self-reported data was conducted in Ethiopia.

## Methods

### Research area and participants

The study was conducted as part of a larger research project investigating handwashing in four Borena kebeles (the smallest administrative units of Ethiopia, similar to wards) in southern Ethiopia. The Borena Zone is a semi-arid region which suffers repeated droughts. Governmental and non-governmental organisations have implemented handwashing interventions in the Borena Zone as part of drought responses since 2006.

Each of the four study kebeles consisted of approximately 30 hamlets, and only those that were reachable by car or a maximum 20-minute walk were included in the study. Within a hamlet, households were selected by the random-route method [40]. The eligibility criterion for participation, which was assessed by self-report, was being the primary caregiver (usually female) to children younger than 5 years old. These individuals were targeted as they are responsible for childcare and cooking and so have the highest potential to transmit diarrhoeal pathogens to the children.

According to sample size estimation with G*Power 3.1 [41], a sample of 300 households would have been necessary to detect a small-to-medium effect at the Type I error probability of 0.05 and a statistical power of 0.95. It was unlikely that all the relevant behaviours (e.g. defecation by the primary caregiver) would occur in each study household during observation sessions. Therefore, to ensure an adequate sample size for the observations of each relevant key time, the researchers aimed to survey 500 randomly selected primary caregivers of children under the age of 5 years. Eventually, 554 primary caregivers were surveyed. Still, the observed data sample sizes were rather small for some key times (e.g. *n* = 71 for the key time of wiping a child’s bottom).

### Research design

The present study was interested in factors associated with over-reporting of handwashing and measures to mitigate it. This focus necessitated a comparison of observed and self-reported data, and to maximise comparability, a cross-sectional correlational design measuring all the variables at one point in time seemed appropriate.

### Data collection procedure

Data collection took place in February and March 2013 through 2.5-hour observations and 1-hour, oral, face-to-face interviews. Observations took place at dawn or around noon during lunch preparation and were followed by interviews. Data were collected by 14 local students and social workers, of whom two were female and 12 male. To ensure high quality in data collection, the team received extensive training in a 4-day workshop. The workshop included an introduction to different question types, demonstrations of how to ask the different question types, a discussion of what to do and what not to do during interviewing and observations (e.g. changing a question’s wording, commenting, (nonverbally) judging responses or behaviour, and lecturing the participant). In addition, the workshop offered a question-by-question discussion of the questionnaire and extensive practice in conducting the interview and the observational procedure through role playing. To ensure that data collection was conducted as in training, the first author, a local research collaborator and a postgraduate student supervised the team during data collection.

### Ethics statement

This study was conducted in strict compliance with the ethical principles of the American Psychological Association (APA; http://www.apa.org/ethics/code/) and the World Medical Association (WMA) Declaration of Helsinki (http://www.wma.net/en/30publications/10policies/b3/). It was part of a larger research project on handwashing which received ethical approval from the Ethiopian National Research Ethics Review Committee and the Ethics Committee of the Philosophical Faculty of University of Zurich. Following approval from these ethics boards, oral informed consent was obtained from all study participants. Written consent could not be obtained because of the high illiteracy rate in the sample. When a selected household refused to participate in the study, the household members were thanked, and the research team members left immediately. The number of refusing households was recorded in a dedicated space in the questionnaire of the next consenting household.

### Measures

The interviews were based on a structured questionnaire developed for this study. The items covered socio-demographic characteristics, self-reported handwashing (standard measure), potential explanatory factors and alternative self-report measures of handwashing. Most of the response options used Likert scales (5-point scales for unipolar items and 9-point scales for bipolar items), which were transformed into a value range of 0–1 (or -1–1 for bipolar items) to facilitate interpretation. The questionnaire was prepared in English, translated into Afan Oromo and then retranslated into English to ensure the quality of the translation. A structured format was prepared for observations [42]. The applicability of the questionnaire and the observation format was verified in a pre-test of *N* = 28. The acquired data are available from the figshare database (DOI 10.6084/m9.figshare.1304955).

### Self-reported handwashing (standard measure—action-based prompted recall)

Self-reported handwashing was measured by nine items using the following format: ‘In general, how often do you wash your hands with soap *before eating*/*after defecation*?’ (0 = *almost never/0–1 out of 10 times* to 1 = *almost always/9–10 out of 10 times* [43, 44]). The key times investigated were those usually promoted in handwashing interventions focusing on diarrhoea prevention [45]: after defecation (and urination), after wiping a child’s bottom or after other contact with stool (stool-related handwashing, SRH); before preparing food or handling drinking water, before feeding or breastfeeding a child and before eating (food-related handwashing, FRH). (During observations, it was clear when caregivers went to defecate or urinate as they left the compound and squatted behind a nearby bush. However, it was not possible to distinguish between defecation and urination. To maintain the comparability of self-reported and observed data, we asked about handwashing not only after defecation but also after urination). Previous research has found that stool- and food-related handwashing are statistically separable and partly explained by different social-cognitive factors [44, 46]. This finding was confirmed in the present study by confirmatory factor analysis. Internal consistency was satisfactory (Cronbach’s α SRH = 0.90, Cronbach’s α FRH = 0.90). Most analyses were run separately for stool- and food-related handwashing as it was assumed that over-reporting and its causative factors might differ at various key times. Separate analyses for each key time were not feasible as the sample sizes for each key time were often too small for observational data.

### Observed handwashing

The same key times measured by self-reports were observed. If a key event occurred, it was noted in a structured format, along with whether both hands were washed with water before or after the event separately and whether soap was used. During data processing, for each respondent and for each type of key event (e.g. food preparation) the percentage of times a participant washed both hands with soap at a key event out of all the times the key event occurred was calculated. To allow for direct comparison with self-reported data, observational data concerning faeces and food were averaged to form observed stool- and food-related handwashing, although internal consistency was rather low (Cronbach’s α stool = 0.46; Cronbach’s α food = 0.56). However, these estimates were based on very small samples (*n*_stool_ = 24 and *n*_food_ = 17) as only a few participants were observed at *all* respective key times; therefore, internal consistency could not be conclusively assessed. Response scales ranged from 0 = *0% handwashing* to 1 = *100% handwashing*.

### Over-reporting in self-reported stool- and food-related handwashing

To examine over-reporting in self-reported handwashing, the observation scores were subtracted from the self-report scores for each individual and separately for stool- and food-related handwashing. The same was done separately for each key time. The response scales ranged from -1 = *100% underreporting* to 0 = *accurate reporting* to 1 = *100% over-reporting*.

### Factors potentially involved in socially desirable responding

#### Socially desirable responding as a personality characteristic

A socially desirable response style was assessed using the short form C [47]of the Marlow—Crown Social Desirability Scale [24]. A previous study verified the Marlow—Crown Social Desirability Scale’s applicability in Ethiopia [48]and the short form has been applied successfully in other African countries [49]. The short form contains 13 items on culturally acceptable and approved behaviours which are relatively unlikely to occur, such as ‘No matter who I’m talking to, I’m always a good listener’ (0 = *false* and 1 = *true*; [24]). To preclude the influence of acquiescence tendency, for some items the socially desirable response was ‘true’ and for others ‘false’. To ensure that all responses were keyed in the format 0 = *no socially desirable response* and 1 = *socially desirable response*, eight items were recoded during data processing. Although internal consistency was low (Cronbach’s α = 0.50), the items were averaged according to the original short form C [47].

#### Need for conformity

This factor was assessed with one item: ‘In general, do you want to do what people who are important to you think you should do?’ (0 = *not at all* to 1 = *very much*; [50]).

#### Group attachment

The item for this factor asked, ‘Do you feel a sense of belonging when you are with members of your community?’ (0 = *no sense of belonging* to 1 = *a strong sense of belonging*; [51]).

#### Social norms

In line with previous research on social norms, descriptive and injunctive norms were considered [52]. To measure the *descriptive norm*, two items tested both stool- and food-related handwashing, including ‘How many people of your community wash their hands with water and soap *before handling food/after contact with faeces*?’ (0 = *almost nobody* to 1 = *almost all of them*; Cronbach’s α stool = 0.75; Cronbach’s α food = 0.79; [46]). The same approach (two items for both stool- and food-related handwashing) was used to measure the *injunctive norm*. Respondents were asked, for example, ‘Do people who are important to you think that you should or should not wash your hands with soap and water *before handling food/after contact with faeces*?’ (-1 = *nearly all think I should not* to 1 = *nearly all think I should*; Cronbach’s α stool = 0.55; Cronbach’s α food = 0.60; [46]).

#### Presence of a spouse or other adults

Immediately before assessing self-reported handwashing, the interviewer observed and recorded whether the spouse (who was in all cases the husband) or other adults were present using the following format: 0 = *spouse not present*, 1 = *spouse present*, 0 = *no other adults present* and 1 = *other adults present*.

### Factors potentially involved in encoding and recall

#### Regular daily household routine

This factor was assessed by asking, ‘During the day, you have to do many things like preparing food and taking care of children and animals. Do you carry out these things in the same order each day?’ (0 = *no* and 1 = *yes*).

#### Frequent task interruptions

First, participants were asked whether they regularly carried out each of five tasks (feed a child, change a baby’s nappy, prepare food, clean dishes and clean the goat shed). Then, for each task participants carried out regularly, they were asked, ‘While you are carrying out the task, how often does something interrupts you in carrying out the task, such as another task, another person or an animal?’ (0 = *never* to 1 = *(almost) always*; Cronbach’s α = 0.82).

#### Estimation tendency

To assess participants’ general estimation tendency (accurate, under- or over-estimation), they were asked to estimate the frequency of an unrelated event. During the interview, participants were asked, ‘How are you feeling?’ five times. At the end of the interview, participants were asked, ‘During the interview, how many times did I ask you how you are feeling?’, and the stated number was recorded. Responses less than 5 indicated a tendency to under-estimate, 5 a tendency to estimate accurately and higher than 5 a tendency to over-estimate.

### Factors potentially involved in cognitive dissonance processes

#### Handwashing knowledge

This factor was measured by asking, ‘Can you tell me how you can prevent getting diarrhoea?’ [46]. If a respondent mentioned a key time for handwashing, 1 point was assigned. Points were added separately for stool- and food-related key times and rescaled to 0 = *no handwashing key time mentioned* to 1 = *all handwashing key times mentioned*.

#### Rationalisation

Rationalisation was measured with six items based on previous research (e.g. [36]), such as ‘Diarrhoea is not as dangerous as handwashing promotions claim’ (-1 = *strongly disagree* to 1 = *strongly agree;* Cronbach’s α = 0.72).

#### Interaction term of handwashing knowledge and rationalisation

The interaction between health knowledge and rationalisation was calculated by separately multiplying the corresponding items (handwashing knowledge and rationalisation) for stool- and food-related handwashing. Items were not centred as both had a meaningful zero point, namely, no health knowledge and a neutral position regarding rationalisation [53].

### Script-based covert recall

Script-based covert recall was measured as follows. Short sequences of daily routines representing handwashing key times were presented to participants, who were asked to explain how they usually carried out these routines in as much detail as possible. The following is an example item:

*Imagine that you have just finished feeding the goats*. *Now your child is hungry*, *and you have to feed your child*. *Please describe exactly what you do from the moment you leave the goat shed until you feed your child*.

Data collectors recorded whether the participants mentioned handwashing with soap in the descriptions of their routines. Four items assessed stool-related handwashing (Cronbach’s α = 0.81) and five items were applied for food-related handwashing (Cronbach’s α = 0.86). The scores for these items were later averaged. Sum scores ranged from 0 = *handwashing with soap mentioned in 0%* to 1 = *handwashing with soap mentioned in 100%* of the descriptions of routines.

### Over-reports in script-based covert recall of stool- and food-related handwashing

Over-reporting in script-based covert recall was calculated in the same way as over-reporting in self-reporting. The observation scores were subtracted from the script-based covert recall scores for each individual and separately for stool- and food-related handwashing. The response scale ranged from -1 = *100% underreporting* to 0 = *accurate reporting* to 1 = *100% over-reporting*.

### Data analysis

All analyses were performed using IBM SPSS Statistics 22 released in 2013. To test whether socially desirable responding and other factors were associated with over-reporting of handwashing (H1–H9; Q1–Q2), Pearson correlations and point-biserial correlations were calculated. H10 regarding the interaction between health knowledge and rationalisation was tested with partial correlations, controlling for health knowledge and rationalisation. All analyses were conducted separately for stool- and food-related handwashing. Respondents with the score of 1 in observed stool- or food-related handwashing (i.e. full handwashing compliance) were excluded from the analyses for the following reasons: (1) no over-reporting was possible for respondents with full observed compliance as the self-report measure scale ended with the category of full compliance; (2) some hypotheses were restricted to respondents with imperfect compliance (H8–H10); (3) some factors were assumed not only to explain over-reporting but also to cause behaviour (i.e. social norms), and thus, these factors were expected to be high among respondents who over-reported, as well as those who fully complied with the observations, that is, respondents who could not over-report.

To investigate whether other factors beyond socially desirable responding might explain over-reporting, four multiple regression models were run separately for stool- and food-related handwashing. Only factors which had significant correlations with over-reporting were considered. The first model (Model A) tested the amount of variance in over-reporting explained by socially desirable responding factors. The second model tested whether the inclusion of factors related to encoding and recall explained additional variance in over-reporting. The third model tested whether the addition of dissonance factors to Model A increased the amount of explained variance in over-reporting. The fourth model (full model) tested the increase in explained variance in over-reporting when all the additional variables (encoding, recall and dissonance) were added to Model A. To increase estimation accuracy, the regression models were tested using bootstrap estimation with 10,000 resamples. Again, respondents with the score of 1 in observed stool- or food-related handwashing (full handwashing compliance) were excluded from the analyses.

Whether the script-based covert recall, which was thought to reduce the influence of recall errors and socially desirable responding, mitigates the response bias (H11) was assessed with dependent *t*-tests that separately compared script-based covert recall to action-based prompted recall (standard self-reported handwashing) for stool- and food-related handwashing. Bootstrapping estimation with 10,000 resamples was applied.

## Results

### Socio-demographic characteristics and preliminary analyses

All of the surveyed primary caregivers were women, and most were married (*n* = 501; 90.4%). While the majority were mothers of a child younger than 5 years old in the household (*n* = 471; 85.2%), a substantial number were grandmothers of a child younger than 5 in the household (*n* = 76; 13.7%). Due to this substantial minority in the sample, the median age of the population was high, and the age range wide (*Mdn* = 30.00 years old; min = 15 years old, max = 96 years old). The vast majority of participants had never attended school (*n* = 535; 96.6%) and were illiterate (*n* = 542; 98.2%). Most of the primary caregivers held traditional beliefs (*n* = 518; 94.0%), and all belonged to the Borena ethnic group. On average, study households consisted of five people (*M* = 5.21, *SD* = 1.90), including one child younger than 5 years old (*M* = 1.35, *SD* = 1.05). The main household livelihood was pastoralism (*n* = 543; 98.0%), and the median daily income per person was US$0.14 (min = US$0.00, max = US$0.85), far below the poverty line of US$1.25 [54].

Of participants, 235 were observed at least at one stool-related key time (e.g. defecation). Of these, only approximately a fifth washed their hands at all the observed key times (*n* = 46, 19.6%), while three-quarters never washed their hands (*n* = 177; 75.3%). Of the 542 participants who were observed at least at one food-related key time (e.g. food preparation), only a small minority washed their hands at all observed key times (*n* = 16; 3%). The vast majority never washed their hands (*n* = 431; 79.5%). To get a clearer picture of the study sample, we compared participant groups based on socio-demographic characteristics. First, we compared participants who consistently washed their hands at *all* stool- or food-related key times with those who inconsistently washed their hands (washed their hands at only some or none of the key times; see Tables A—D in S1 File). Second, we compared participants who consistently washed their hands at *all stool-related* key times with those who consistently washed their hands at *all food-related* key times (see Tables E and F in S1 File). Participant groups differed in only one socio-demographic characteristic: household size. Primary caregivers who washed their hands at all *food-related* key times belonged to larger households than (1) those who did not wash their hands at all food-related key times (see Table C in S1 File) and (2) those who washed their hands at all stool-related key times (see Table E in S1 File). The interested reader can refer to S1 File which presents the applied methods and detailed results.

In contrast to observed handwashing, self-reported handwashing at key times was high (see Table 1). Over-reporting of handwashing at key times was also high, with mean values between *M* = 0.47 and *M* = 0.63. These results mean that respondents reported approximately 50%–60% more handwashing than they actually performed. Over-reporting was higher for food- than stool-related key times. The highest mean value was found for handwashing before eating, while the lowest mean value was found for handwashing after defecation or urination. Self-reported and observed handwashing were significantly correlated for all stool-related key times. For food-related key times, however, the two measures had significant correlations at only two key times: before preparing food and before breastfeeding a child.

**Table 1:** Descriptive statistics and Pearson correlations for self-reported and observed handwashing and descriptive statistics for over-reporting at key times.

		*SRHW*		*OHW*			*OvR*	
Key time	*N*	*M*	*SD*	*M*	*SD*	*r SRHW-OHW*	*M*	*SD*
*Stool-related key times (mean)*	235	0.72	0.26	0.22	0.40	.27***	0.48	0.42
After defecation or urination	190	0.68	0.30	0.21	0.40	.32***	0.47	0.42
After wiping a child’s bottom	71	0.73	0.31	0.23	0.42	.28**	0.50	0.45
After other kinds of contact with stool	62	0.70	0.28	0.19	0.40	.24*	0.50	0.43
*Food-related key times (mean)*	542	0.66	0.25	0.10	0.23	.17***	0.57	0.33
Before eating	305	0.70	0.28	0.07	0.26	-.02	0.63	0.39
Before preparing food	420	0.69	0.28	0.17	0.36	.22***	0.52	0.41
Before breastfeeding a child	207	0.66	0.31	0.06	0.21	.19**	0.60	0.34
Before feeding a child	337	0.68	0.29	0.08	0.26	.05	0.61	0.38
Before handling drinking water	225	0.60	0.30	0.10	0.29	.05	0.50	0.41

*Note*. SRHW = Self-reported handwashing. OHW = Observed handwashing. OvR = Over-reporting.

*** *p* ≤ 0.001.

** *p* ≤ 0.01.

* *p* ≤ 0.05. One-tailed significance levels are presented.

### Factors associated with over-reporting

To answer the first global research question of whether socially desirable responding and additional factors are associated with over-reporting of handwashing, Table 2 presents the correlations between over-reporting and potentially associated factors. Regarding socially desirable responding, Marlow—Crown Social Desirability Scale scores, need for conformity and descriptive and injunctive norms were all positively correlated with over-reporting of stool- and food-related handwashing, supporting H1, H2 and H4. However, contrary to hypothesis H3, a feeling of affiliation was not related to over-reporting of stool- or food-related handwashing. As well, the presence of neither a spouse nor other adults was related to over-reporting of stool- or food-related handwashing, negating Q1 and Q2.

**Table 2:** Descriptive statistics and Pearson correlations for stool-related over-reporting (below diagonal) and food-related over-reporting (above diagonal) with predictor variables.

Variable	OvR	MCSDS	NC	FOA	DN	IN	PS^a^	PA^a^	RDR^a^	FTI	ET	HK	RA	*M* ^*b*^	*SD*
OvR		**0.18**	**0.15**	0.04	**0.45**	**0.31**	-0.03	-0.02	0.04	**-0.14**	**0.16**	**0.18**	**-0.31**	0.59	0.28
MCSDS	**0.24**		-0.**04**	-0.07	**0.24**	**0.10** ^**c**^	0.02	0.03	**0.09** ^**d**^	**0.20**	0.02	**0.19**	0.04	0.56	0.18
NC	**0.17** ^**c**^	-0.04		**0.35**	**0.21**	**0.31**	-0.04	-0.02	**0.10** ^**c**^	**-0.09** ^**d**^	**0.10** ^**d**^	**0.15**	**-0.19**	0.85	0.15
FOA	-0.11	-0.06	**0.42**		**0.12** ^**c**^	**0.17**	0.01	-0.01	**0.15**	**-0.12** ^**c**^	**0.13** ^**c**^	**-0.08** ^**d**^	**-0.13** ^**c**^	0.85	0.14
DN	**0.58**	**0.25**	**0.15** ^**c**^	-0.01		**0.41**	-0.01	-0.02	0.02	**-0.14**	**0.18**	**0.18**	**-0.34**	0.66	0.23
IN	**0.43**	0.01	**0.36**	**0.16** ^**d**^	**0.46**		0.06	**0.09** ^**d**^	0.06	0.06	**0.15**	**0.09** ^**d**^	**-0.23**	0.83	0.19
PS^a^	-0.07	-0.02	-0.06	0.00	0.02	0.01		-0.06	0.04	**0.17**	-0.04	0.02	-0.01	32%	
PA^a^	0.02	0.02	0.00	-0.01	0.04	**0.16** ^**d**^	-0.09		0.00	-0.01	-0.03	0.04	**0.09** ^**d**^	18%	
RDR^a^	**-0.12** ^**d**^	-0.02	0.09	**0.14** ^**d**^	0.00	-0.02	0.07	-0.07		-0.04	**-0.08** ^**d**^	-0.03	**-0.08** ^**d**^	77%	
FTI	**-0.14** ^**d**^	**0.20** ^**c**^	-0.03	-0.01	-0.04	0.10	0.10	0.03	0.03		-0.05	-0.02	**0.36**	37	0.22
ET	**0.17** ^**c**^	0.03	**0.16** ^**d**^	**0.17** ^**c**^	**0.24**	**0.22**	-0.01	-0.05	0.01	-0.06		**0.08** ^**d**^	**-0.17**	5.55	3.82
HK	0.02	0.10	0.08	0.07	**0.19** ^**c**^	0.10	-0.02	0.04	0.09	**0.26**	-0.04		**-0.28**	0.20	0.29
RA	**-0.33**	0.01	**-0.19** ^**c**^	-0.10	**-0.40**	**-0.23**	-0.09	0.11	-0.10	**0.30**	**-0.22**	-0.05		-0.40	0.43
*M* ^*b*^	0.66	0.56	0.83	0.84	0.65	0.79	30%	20%	76%	0.39	5.49	0.31	-0.30		
*SD*	0.26	0.17	0.16	0.14	0.22	0.24				0.23	4.27	0.29	0.45		

*Note*. *N* stool-related over-reporting = 189. *N* food-related over-reporting = 525. OvR = Over-reporting; MCSDS = Marlow—Crown Social Desirability Scale; NC = Need for conformity; FOA = Feeling of affiliation; DN = Descriptive norm; IN = Injunctive norm; PH = Presence of spouse; PA = Presence of adults; RDR = Regular daily routine; FTI = Frequent task interruptions; ET = Estimation tendency; HK = Health knowledge; RA = Rationalisation.

^a^ Correlations are point-biserial correlations.

^b^ For the dichotomous variables of PS, PA and RDR, percentages are presented, instead of mean.

Boldface: significant with *p* ≤ 0.001, except for the following:

^c^ *p* ≤ 0.01;

^d^ *p* ≤ 0.05. One-tailed significance levels are presented, except for PS and PA.

With regards to factors affecting encoding and recall, a regular daily household routine was negatively correlated with over-reporting of stool- but not food-related handwashing, partly supporting H5. Frequent task interruption was correlated with over-reporting of stool- and food-related handwashing; however, against hypothesis H6, the correlations were negative. Participants’ estimation tendencies were positively correlated with over-reporting of both stool- and food-related handwashing, supporting H7.

For dissonance processes, health knowledge was positively related to over-reporting of food- but not stool-related handwashing, partly supporting H8. In line with H9, rationalisation was negatively correlated with over-reporting of both stool- and food-related handwashing. Based on the partial correlations of over-reporting with the interaction term of health knowledge and rationalisation, while controlling for health knowledge and rationalisation, health knowledge and rationalisation did not interact in over-reporting of stool- and food-related handwashing (*r*_stool_ = -0.03, *p* = 0.346; *r*_food_ = 0.03, *p* = 0.273). H10, thus, was not supported.

The variables significantly associated with over-reporting were incorporated in the subsequent regression models. These variables included frequent task interruptions, although, contrary to expectations, this factor was negatively associated with over-reporting.

### Factors beyond socially desirable responding explaining over-reporting

With regards to the second global research question of regarding whether additional factors beyond socially desirable responding explain over-reporting, socially desirable responding factors significantly explained variance in over-reporting of stool- and food-related handwashing (see Model A in Tables 3 and 4). After encoding and recall factors were added, the explained variance increased significantly for over-reporting of both stool- and food-related handwashing (see Model A+1 in Tables 3 and 4). The explained variance in over-reporting of stool- and food-related handwashing also increased when adding dissonance factors to the socially desirable responding factors. However, the increase was significant for over-reporting of food- but not stool-related handwashing (see Model A+2 in Tables 3 and 4). When adding all additional factors (encoding, recall and dissonance) to the socially desirable responding factors, the increase in explained variance was significant for over-reporting of both stool- and food-related handwashing (see Model A+3 in Tables 3 and 4).

**Table 3:** Factors explaining over-reporting in stool-related handwashing: Multiple regression results.

				Model A+3 (full model)	
Variable	Model A (SDR only) *B*	Model A+1 (encode/recall) *B*	Model A+2 (dissonance) *B*	*B*	90% CI
Constant	-0.03	0.06	-0.01	0.08	[-0.09, 0.24]
MCSDS	0.19*	0.25**	0.21**	0.26**	[0.11, 0.41]
Need for conformity	0.05	0.05	0.03	0.04	[-0.12, 0.21]
Descriptive norm	0.54***	0.50***	0.48***	0.47***	[0.33, 0.61]
Injunctive norm	0.24	0.27***	0.24**	0.27***	[0.14, 0.39]
Regular daily routine	-	-0.07*	-	-0.07**	[-0.13, -0.02]
Frequent task interrupt.	-	-0.20**	-	-0.17**	[-0.29, -0.06]
Tendency to over-estimate ^a^	-	0.00	-	0.00	[-0.01, 0.01]
Rationalisation	-	-	-0.07*	-0.05†	[-0.11, 0.01]
*R* ^*2*^	.39	.43	.40	.44	
*F*	29.69***	19.86***	24.83***	17.69***	
Δ *R* ^*2*^ compared to Model A		.04	.01	.05	
Δ *F* compared to Model A		4.50**	3.69†	3.86**	

*Note*. *N* = 189. CI = Confidence interval. SDR = Socially desirable responding. MCSDS = Marlow—Crown Social Desirability Scale.

*** *p* ≤ 0.001.

** *p* ≤ 0.01.

* *p* ≤ 0.05.

^†^ *p* ≤ 0.10. One-tailed significance levels are presented, except for the constant and frequent task interruptions.

^a^ The answer scale ranged from 0 to infinite.

**Table 4:** Factors explaining over-reporting in food-related handwashing: Multiple regression results.

				Model A+3 (full model)	
Variable	Model A (SDR only) *B*	Model A+1 (encode/recall) *B*	Model A+2 (dissonance) *B*	*B*	90% CI
Constant	-0.01	0.03	0.02	0.04	[-0.08, 0.15]
MCSDS	0.12*	0.17**	0.14*	0.16**	[0.07, 0.28]
Need for conformity	0.06	0.04	0.02	0.01	[-0.10, 0.14]
Descriptive norm	0.46***	0.41***	0.38***	0.36***	[0.28, 0.45]
Injunctive norm	0.21***	0.23***	0.19**	0.20**	[0.10, 0.30]
Frequent task interrupt.	-	-0.16**	-	-0.10*	[-0.19, -0.01]
Tendency to over-estimate ^a^	-	0.01†	-	0.00	[0.00, 0.01]
Health knowledge	-	-	0.05†	0.05†	[-0.07, 0.11]
Rationalisation	-	-	-0.11***	-0.09**	[-0.14, -0.04]
*R* ^*2*^	.23	.25	.26	.27	
*F*	39.02***	28.82***	30.45***	23.75***	
Δ *R* ^*2*^ compared to Model A		.02	.03	.04	
Δ *F* compared to Model A		6.72**	10.49***	6.75***	

*Note*. *N* = 526. CI = Confidence interval. SDR = Socially desirable responding. MCSDS = Marlow—Crown Social Desirability Scale.

^†^ *p* ≤ 0.10.

*** *p* ≤ 0.001.

** *p* ≤ 0.01.

* *p* ≤ 0.05. One-tailed significance levels are presented, except for the constant and frequent task interruptions.

^a^ The answer scale ranged from 0 to infinite.

In this last model, over-reporting of stool-related handwashing was significantly explained by three socially desirable responding factors (Marlow—Crown Social Desirability Scale scores and descriptive and injunctive norms) and two encoding and recall factors (regular daily routine and frequent task interruptions). The dissonance factor of rationalisation only marginally explained over-reporting of stool-related handwashing. Regarding over-reporting of food-related handwashing, the same socially desirable responding factors (Marlow—Crown Social Desirability Scale scores and descriptive and injunctive norms), one encoding and recall factor (frequent task interruptions) and one dissonance factor (rationalisation) significantly explained over-reporting, while the dissonance factor of knowledge only marginally explained over-reporting. As in all models, the explained variance was higher in over-reporting of stool- than food-related handwashing. Still, for both full models, the effect sizes were large (*f* ^2^_stool_ = .78; *f* ^2^_food_ = .37; cf. [54]).

### Alternative self-report measure to mitigate over-reporting: Script-based covert recall

Our last global research question was whether an alternative self-report measure designed to reduce the influence of socially desirable responding and additional factors mitigates the response bias. Supporting H11, over-reporting was smaller for script-based covert recall compared to standard self-reports (action-based prompted recall). Over-reporting of stool-related standard self-reports (*M* = 0.50, *SD* = 0.41) were significantly larger than over-reporting of stool-related script-based covert recall (*M* = 0.16, *SD* = 0.50); *t*(231) = 11.72, *p* < 0.001, *d* = 0.78. The same pattern holds for over-reporting of food-related standard self-reports (*M* = 0.56, *SD* = 0.31) compared to over-reports in food-related script-based covert recall (*M* = 0.24, *SD* = 0.43); *t*(541) = 19.67, *p* < 0.001, *d* = 0.88. However, the standard deviations of over-reporting were larger in script-based covert recall than in standard self-reports. In other words, script-based covert recall increased response accuracy on the aggregate but not the individual level.

## Discussion

The present study aimed to better understand the causes and underlying mechanisms of over-reporting of handwashing in order to identify indications of measures to mitigate response bias. Socially desirable responding and factors involved in the encoding and recall of information and in dissonance processes were investigated as potential explanatory variables. The mitigating capacity of an alternative self-report measure designed to reduce the influence of socially desirable responding and recall errors was tested. In the following, we first discuss our findings regarding factors associated with over-reporting.

### Factors associated with over-reported handwashing

Overall, over-reporting was quite high, especially for food-related key times. With regards to the first global research question, several factors thought to increase socially desirable responding were correlated with over-reporting of both stool- and food-related handwashing. First, a socially desirable response style was positively associated with over-reported handwashing, in line with previous research that found associations with self-reported hygiene behaviour [21] and other preventive health behaviours [25]. Second, over-reporting was positively correlated to injunctive and descriptive norms. This finding corresponds with the view that socially desirable responding depends on the sensitivity or normative loading of a question [23]. The finding is also in line with previous research that found an association between descriptive norms and socially desirable answers about election participation and cheating [27]. However, the presence of a spouse or other adults during the interview, which could increase or decreasing socially desirable responding to sensitive questions, was not associated with over-reporting of either stool- or food-related handwashing. Previous research on this subject is mixed, indicating that the presence of third parties led to responses that were sometimes *more* socially desirable, *less* socially desirable or uninfluenced (e.g. [29]). It is possible that, in the present project, the presence of third parties did not influence responses as personal privacy and confidentiality usually are neither common nor highly valued in developing countries, especially in rural areas [55]. Alternatively, the influence of the presence of a stranger (the interviewer) on the responses might have been so high to negate the influence of third parties.

Factors assumed to cause response bias through encoding and recall processes were partly associated with over-reported handwashing but not always as hypothesised. First, a regular daily routine was negatively correlated with stool-related over-reporting and uncorrelated to food-related over-reporting. The hypothesis that a regular daily routine facilitates remembering to perform handwashing and leads to more accurate behaviour encoding and reporting, thus, was only partly supported. The influence of a regular daily routine on over-reporting of handwashing should be further investigated, preferably by using a multi-item interval scaled measure, instead of the applied single-item dichotomous measure. Second, frequent task interruptions were associated with over-reporting of both stool- and food-related handwashing. However, contrary to the hypothesis H6, the association was negative, meaning that respondents whose tasks were frequently interrupted tended to over-report less than those who were rarely interrupted. Possibly, frequently interrupted respondents were, against expectations, *less* likely to forget handwashing as interruptions forced them to start and perform their key activities more consciously. Third, over-reporting of stool- and food-related handwashing was also correlated with participants’ general estimation tendency: compared to a person who did not over-estimate the frequency of an unrelated event, a person who did over-estimate had a greater tendency to over-report handwashing. This pattern parallels the finding that individuals’ memory performance typically differs [33, 34].

Finally, over-reporting was also partly associated with factors assumed to affect responses by dissonance processes. According to expectations, rationalisation was negatively correlated with both stool- and food-related over-reporting. Health knowledge, however, was positively correlated with food- but not stool-related over-reporting. This result partly supports the notion that experiencing dissonance not only might result in behaviour change and rationalisation [35]but might also cause *over-reporting* (or underreporting) to restore consonance [27, 37].

Unnoticed forgetting of handwashing, which was assumed to contribute significantly to over-reporting of handwashing, was tested only indirectly through the factors of regular daily routine and frequent task interruptions. To determine whether forgetting affects handwashing frequency as outlined and whether it adds to the bias caused by socially desirable responding, over-reporting of handwashing could be compared in a future study with over-reporting of another preventive health behaviour which is equally socially desirable but for which forgetting and other encoding and recall errors are thought to be less pronounced, such as solar water disinfection.

Overall, socially desirable responding processes (socially desirable response style, need for conformity and injunctive and descriptive norms), encoding and recall factors (regular daily routine, frequent task interruptions and general estimation tendency) and dissonance processes (health knowledge and rationalisation) were associated with over-reported handwashing. These additional factors explained over-reporting of handwashing beyond socially desirable responding. This new knowledge about explanatory factors for over-reported handwashing could be used to develop measures to mitigate the response bias in self-reported handwashing by reducing the influence of these factors. Three approaches have been proposed to alleviate the influence of factors which cause a response bias [23, 56]: (1) statistical control of causative factors; (2) optimisation of the interview situation; and (3) improvement of self-report measures. Appropriate mitigation measures are discussed in the following section.

### Measures to mitigate over-reported handwashing

#### Statistical control of causative factors

It has been suggested to control for the influence of a socially desirable response style by partialling out its effect through multiple regression analysis or partial correlations [56]. This method could be applied for all factors found to be associated with over-reported handwashing. However, some of these explanatory factors (social norms and health knowledge) are also thought to determine behaviour (including handwashing; [35, 57]), so statistical control is not possible for these factors. The remaining factors (socially desirable response style, need for conformity, regular daily routine, frequent task interruptions, general estimation tendency and rationalisation) had mediocre explanatory power for over-reporting, so applying this method for them would result in negligible improvement to the accuracy of self-reports.

#### Optimisation of the interview situation to reduce socially desirable responding

Instead of controlling for socially desirable responding *after* data collection, it could be controlled *during* data collection by optimising the survey situation. First, the perceived privacy or confidentiality during data collection could be increased [23]. The present study found that the presence or absence of acquainted third parties during the interview seems irrelevant in social and cultural contexts similar to the one investigated. The influence of the presence of a stranger (the interviewer), although not tested here, might have a major influence on over-reporting. While self-administered questionnaires are often not feasible in developing countries because of high illiteracy, interviewers could only read closed questions while respondents record their answers themselves with rating scales using symbolic labels (e.g. smileys or minus and plus signs), instead of words [58]. Such an approach should be tested in future research.

Second, as has been successfully done in previous studies, the bogus pipeline procedure could be applied to reduce socially desirable responding. In this method, respondents are made to believe that the interviewers will learn their true behaviour regardless of self-reports because an additional measure (e.g. a microbiological hand contamination assay) will also be applied [23]. It could be interesting to test the practicability of this method in future studies. Alternatively, one could attempt to inform respondents explicitly and extensively about the tendency to give socially desirable responses while emphasising its dangers as the accuracy of information about a community might be lost, and the provision of essential help might be delayed. Respondents then might feel inclined to give accurate responses to serve the needs of their communities.

#### Improved self-report measures

In addition to statistical control and optimised interview situations, improved self-report measures could also lessen the influence of some factors associated with over-reported handwashing. To reduce the effects of socially desirable responding, varying the wording or context of questions has been suggested to encourage honest, potentially embarrassing responses [23]. Only a few studies have tested the ability of such approaches to mitigate socially desirable responding, and the majority of these studies found that these approaches did not successfully prevent socially desirable responding (for an overview, see [23]). As part of the present project, several techniques suggested in the literature were tested, without success. The interested reader is referred to S2 file, which presents the applied methods and results.

The influence of recall errors, in addition to socially desirable responding, was also targeted with an alternative self-report measure, a script-based covert recall. While the mean over-reporting in this script-based covert recall was smaller than in conventional self-reports, the variance in over-reporting was even higher. In other words, the new measure was more accurate on the aggregate but not the individual level, probably because of frequent *under*-reporting. It is possible that handwashing, especially for habitual hand washers, is such a natural component of these situations that it did not seem worth mentioning.

Some alternative instruments which are thought to increase recall accuracy were not applied in the present research. First, 24-hour recalls, often used in dietary intervention studies (e.g. [59]), could be applied. In these, respondents would be asked to recall in reverse the performed activities relevant to handwashing (e.g. food preparation) in the past 24 hours, and the time and occasion of these events could serve as cues to facilitate remembering. Second, weekly recall diaries might be used [60]. In these, respondents could be provided with a structured form and asked to record each evening over a week how many times they performed each key event and at how many they had washed their hands. While both measures are thought to increase recall accuracy, they do not mitigate socially desirable responding or encoding errors. Furthermore, respondents would be more aware of handwashing in the latter measure, so it might cause reactivity and thus change behaviour. Still, the applicability of these measures could be tested in future studies and perhaps combined with the outlined approaches to mitigate socially desirable responding (see section Optimisation of the interview situation to reduce socially desirable responding).

### Study strengths, limitations and perspectives

Although the difficulties involved in measuring handwashing by self-reporting are widely known (e.g. [11–13]), this is the first paper to examine potential explanatory factors for over-reported handwashing. We examined not only socially desirable responding, the factor usually regarded as responsible for the bias [12], but also considered additional factors involved in the encoding, retrieval and integration of relevant information. Alternative self-report measures expected to mitigate socially desirable responding and/or recall errors were also tested. While none of the applied measures increased response accuracy on the individual level, script-based covert recall increased response accuracy on the aggregate level. Additional research is required to verify the mitigating capacity of the script-based covert recall and to test further alternative measures which were discussed.

The present study assessed over-reporting directly by subtracting observed from self-reported behaviour scores. Consequently, we could investigate the explanatory factors’ *actual* effects on over-reporting in isolation from the influence that the factors (e.g. social norms) might have also had on actual behaviour and thus responding. Whether observed behaviour is the most valid measure for actual behaviour and, thus, the standard of comparison for other measures can be questioned. However, answering this question was not within the scope of the present study.

In the present study, care was taken to ensure the comparability of the measures used for observed and self-reported behaviour to the greatest extent possible. Self-reported and observed behaviour were measured on the same day, and comparable response scales in interviews and recording formats in observations were employed. While the worth of comparability seems rather basic, previous studies that compared self-reported and observed behaviour used answer scales and observation formats that were not comparable (e.g. [13, 17, 19]) and self-reports and observations measured on different days, even up to two months apart (e.g. [17–19]).

Still, some factors limited comparability in the present study. Observations lasted for only 2.5 hours because the usual daily routines of primary caregivers in the studied region rarely left them at home for more than 3–4 hours at a time. Within 2.5 hours, repeated observation of one key time (e.g. food preparation) was rare. Accordingly, handwashing compliance at a specific key time was often determined by a single observation event. Self-reports, in contrast, asked for an integrated estimation of handwashing frequency at a key time. Future studies investigating over-reporting of handwashing should attempt to further increase the comparability between self-reported and observation measures.

A further limitation of the present study is that some factors were tested even though the internal consistency of their measures was insufficient (i.e. Marlow—Crown Social Desirability Scale). The internal consistency of the observation measures was also not conclusively assessed. The internal consistency of these measures requires further investigation.

Several of the assumed underlying mechanisms were not tested directly, in particular, dissonance processes and the influence of unnoticed forgetting of washing hands on over-reporting. In addition to the idea for future research regarding forgetting already mentioned, dissonance processes could be investigated through experimental research testing whether the induction of dissonance (e.g. by providing health information) increases over-reporting (or under-reporting, depending on the health behaviour at hand).

Though many factors were considered in the present paper, several factors were not included even though they might affect over-reporting of handwashing (e.g. courtesy bias, acquiescence tendency, question order). Although some of these are rather unlikely to be relevant given the response patterns found in the present study (e.g. acquiescence tendency), the factors should be investigated in subsequent studies.

An additional aspect which is essential to measurement but was not considered in the present paper is reliability; a measure’s consistency or repeatability. Previous studies have questioned the inter-rater and retest reliability of observed handwashing in both household and hospital settings [14, 61, 62]. Future research should further investigate the reliability of self-reported and observed handwashing by applying repeated measurements. If the reliability of observations is found to be low, it would raise doubts about the adequacy of using single observation sessions to evaluate handwashing interventions. In addition, self-reports might reflect an integrated behaviour assessment, and a single observation session might be a situation-dependent snapshot in time. Therefore, it might be fruitful to compare self-reported handwashing rates to the rates captured in several observation sessions (with the latter also reflecting a more integrated behaviour assessment).

## Conclusion

The difficulties involved in measuring handwashing by self-reports and observations, including over-reporting of self-reported handwashing, are widely known. The present paper is the first to investigate explanatory factors for over-reporting of handwashing. This study also tested measures to mitigate over-reporting. This research has contributed to the limited evidence base for a complex phenomenon and a highly important subject: how to evaluate handwashing interventions efficiently and accurately. We hope that the present paper stimulates further research on over-reporting of handwashing and on alternative self-report measures to mitigate response bias.

## Supporting Information

S1 File Differences in socio-demographic characteristics of consistent hand washers and inconsistent/non-hand washers. Means (*M*) and standard deviations (*SD*) of socio-demographic characteristics for inconsistent and consistent stool-related hand washers and related *t*-tests (Table A). Frequencies in socio-demographic characteristics for inconsistent and consistent stool-related hand washers and related *χ* ^*2*^-tests (Table B). Means (*M*) and standard deviations (*SD*) of socio-demographic characteristics for inconsistent and consistent food-related hand washers and related *t*-tests (Table C). Frequencies in socio-demographic characteristics for inconsistent and consistent food-related hand washers and related *χ* ^*2*^-tests (Table D). Means (*M*) and standard deviations (*SD*) of socio-demographic characteristics for consistent stool-related hand washers and consistent food-related hand washers and related *t*-tests (Table E). Frequencies in socio-demographic characteristics for consistent stool-related hand washers and consistent food-related hand washers and related *χ* ^*2*^-tests (Table F).
(PDF)

S2 File Alternative self-report measures to mitigate question sensitivity. (PDF)
