# Supplementary material for: Over-Reporting in Handwashing Self-Reports: Potential Explanatory Factors and Alternative Measurements
Source: PLoS One. 2015 Aug 24;10(8):e0136445. doi: 10.1371/journal.pone.0136445 (PMC4547747; doi:10.1371/journal.pone.0136445)
Supplement: S1 File — Means (M) and standard deviations (SD) of socio-demographic characteristics for inconsistent and consistent stool-related hand washers and related t-tests (Table A). Frequencies in socio-demographic characteristics for inconsistent and consistent stool-related hand washers and related χ 2-tests (Table B). Means (M) and standard deviations (SD) of socio-demographic characteristics for inconsistent and consistent food-related hand washers and related t-tests (Table C). Frequencies in socio-demographic characteristics for inconsistent and consistent food-related hand washers and related χ 2-tests (Table D). Means (M) and standard deviations (SD) of socio-demographic characteristics for consistent stool-related hand washers and consistent food-related hand washers and related t-tests (Table E). Frequencies in socio-demographic characteristics for consistent stool-related hand washers and consistent food-related hand washers and related χ 2-tests (Table F). (PDF) [file pone.0136445.s001.pdf]

S1 File:

Differences in socio-demographic characteristics between consistent and inconsistent / non-hand washers

Over-reporting in handwashing self-reports: Potential explanatory factors and alternative measurements

Nadja Contzen[1*], Sandra De Pasquale[2], Hans-Joachim Mosler[1]

[1] Environmental and Health Psychology, Department of Environmental Social Sciences, Eawag: Swiss Federal Institute of Aquatic Science and Technology, Duebendorf, Switzerland

[2] Department of Psychology, University of Zurich, Zurich, Switzerland

[*] Corresponding author

E-mail: nadja.contzen@eawag.ch

Methods

As preliminary analyses, we compared participant groups based on socio-demographic characteristics. First, we compared participants who consistently washed their hands at *all* stool- or food-related key times with those who inconsistently washed their hands at stool- or food-related key times (washed their hands only at some or none of the key times). Second, we compared participants who consistently washed their hands at all stool-related key times with those who consistently washed their hands at all food-related key times. The socio-demographic characteristics considered were marital status, family role, age, education, literacy, religion, household size, main household livelihood, daily income per person and number of children below age 5 in the household. *t*-tests were applied for interval scaled variables and contingency tables with χ^2-tests for categorical variables. For several variables differences between groups were tested, so the family wise error rate was controlled with Bonferroni corrections [1]. The significance level for the whole family of comparisons (e.g. all socio-demographic characteristics of consistent and inconsistent stool-related hand washers) was set at α = .05. The Bonferroni corrected significance level for a single comparison (e.g. age of consistent and inconsistent stool-related hand washers) was calculated using the equation p = .05/n, with n being the number of tested socio-demographic characteristics. With n = 10 the resulting significance level was p = 0.005.

Results

Participant groups differed only in one socio-demographic characteristic: household size. Primary caregivers who washed their hands at all food-related key times were from larger households than those who did not wash their hands at all food-related key times (see Table C) and those who washed their hands at all stool-related key times (see Table E).

Table A. Means (*M*) and standard deviations (*SD*) of socio-demographic characteristics for inconsistent and consistent stool-related hand washers and related t-tests.

	Inconsistent SRH			Consistent SRH			*t*-test	
**Socio-demographic characteristics**	***N***	***M***	***SD***	***N***	***M***	***SD***	***t***	p^a
Age	188	31.99	12.16	46	33.98	14.48	-0.95	0.341
Household size	189	5.11	1.76	46	5.02	1.93	0.30	0.762
Number of children below age 5	186	1.40	0.60	45	1.47	0.92	-0.57	0.571
Daily income per person (US$)	87	0.23	0.22	24	0.14	0.15	2.27	0.028

Note. SRH = Stool-related handwashing.
[a] Bonferonni corrected significance $p < .005$.

Table B. Frequencies in socio-demographic characteristics for inconsistent and consistent stool-related hand washers and related χ^2-tests.

Socio-demographic characteristics		Number of respondents (SR)			χ^2-tests	
		**Inconsistent SRH**	**Consistent SRH**	**Total**	**χ^2 (df)**	**p^a**
Marital status	Married	176 (0.1)	41 (-0.2)	217	0.83 (1)	0.262
	Single/widowed/divorced [b]	13 (-0.4)	5 (0.8)	18		
	Total	189	46	235		
Family role	Mother	166 (0.2)	38 (-0.3)	204	1.16 (2)	0.559
	Grandmother	20 (-0.4)	7 (0.7)	27		
	Sister, aunt, stepmother [b]	2 (-0.3)	1 (0.5)	3		
	Total	188	46	234		
Education	No school attendance	185 (0.2)	41 (-0.5)	226	7.70 (1)	0.016
	School attendance [b]	4 (-1.2)	5 (2.4)	9		
	Total	189	46	235		
Literacy	Neither read nor write	187 (0.1)	44 (-0.2)	231	2.39 (1)	0.173
	Only read/read and write [b]	2 (-0.7)	2 (1.4)	4		
	Total	189	46	235		
Religion	Traditional beliefs	178 (0.0)	43 (-0.1)	221	0.10 (1)	0.723
	Muslim/Christian/other [b]	10 (-0.1)	3 (0.3)	13		
	Total	189	46	234		
Livelihood	Pastoralism	188 (0.0)	45 (-0.1)	233	1.19 (1)	0.354
	Farming + pastoralism/other [b]	1 (-0.5)	1 (-0.5)	2		
	Total	189	46	235		

Note. SR = Standardised residuals SRH = Stool-related handwashing.
[a] Bonferonni corrected significance $p < 0.005$.
[b] The remaining categories were merged into one category due to very low frequencies.

Table C. Means (*M*) and standard deviations (*SD*) of socio-demographic characteristics for inconsistent and consistent food-related hand washers and related *t*-tests.

	Inconsistent FRH			Consistent FRH			*t*-test	
**Socio-demographic characteristics**	***N***	***M***	***SD***	***N***	***M***	***SD***	***t***	**p^a**
Age	525	33.90	13.40	16	36.63	13.15	-0.80	0.423
Household size	526	5.20	1.86	16	6.69	2.44	-3.13	0.002
Number of children below age 5	520	1.37	1.07	15	1.27	0.46	0.36	0.715
Daily income per person (US$)	248	0.20	0.21	7	0.09	0.12	0.13	0.188

Note. FRH = Food-related handwashing.
[a] Bonferonni corrected significance $p < 0.005$.

Table D. Frequencies in socio-demographic characteristics for inconsistent and consistent food-related hand washers and related χ^2-tests.

Socio-demographic characteristics		Number of respondents (SR)			χ^2-tests	
		Inconsistent FRH	Consistent FRH	Total	χ^2 (df)	p^a
Marital status	Married	475 (-0.1)	16 (0.4)	491	1.71 (1)	0.385
	Single/widowed/divorced [b]	51 (0.2)	0 (-1.2)	51		
	Total	526	16	542		
Family role	Mother	450 (0.0)	14 (0.1)	464	0.16 (2)	0.920
	Grandmother	70 (0.0)	2 (-0.1)	72		
	Sister/aunt/stepmother [b]	5 (0.1)	0 (-0.4)	5		
	Total	525	16	541		
Education	No school attendance	508 (0.0)	15 (-0.1)	523	0.37 (1)	0.440
	School attendance [b]	18 (-0.1)	1 (0.6)	19		
	Total	526	16	542		
Literacy	Neither read nor write	514 (0.0)	16 (0.1)	530	0.31 (1)	0.999
	Only read/read and write [b]	10 (0.1)	0 (-0.5)	10		
	Total	524	16	540		
Religion	Traditional beliefs	493 (0.1)	13 (-0.5)	506	4.57 (1)	0.068
	Muslim/Christian/other [b]	30 (-0.4)	3 (2.0)	33		
	Total	523	16	539		
Livelihood	Pastoralism	515 (0.0)	16 (0.1)	531	0.34 (1)	0.999
	Farming + pastoralism/other [b]	11 (0.1)	0 (-0.6)	11		
	Total	526	16	542		

Note. SR = Standardised residuals. FRH = Food-related handwashing.
[a] Bonferonni corrected significance $p < 0.005$.
[b] The remaining categories were merged into one category due to very low frequencies.

Table E. Means (*M*) and standard deviations (*SD*) of socio-demographic characteristics for consistent stool-related hand washers and consistent food-related hand washers and related *t*-tests.

	Consistent SRH			Consistent FRH			*t*-test	
**Socio-demographic characteristics**	***N***	***M***	***SD***	***N***	***M***	***SD***	***t***	p^a
Age	44	33.75	14.19	14	36.29	11.96	-0.60	0.549
Household size	44	5.02	1.92	14	6.93	2.40	-3.04	0.004
Number of children below age 5	43	1.49	0.93	13	1.31	0.48	0.67	0.507
Daily income per person (US$)	22	0.15	0.16	5	0.11	0.14	0.54	0.592

Note. SRH = Stool-related handwashing. FRH = Food-related handwashing.
[a] Bonferonni corrected significance $p < 0.005$.

Table F. Frequencies in socio-demographic characteristics for consistent stool-related hand washers and consistent food-related hand washers and related χ^2-tests.

Socio-demographic characteristics		Number of respondents (SR)			χ^2-tests	
		Consistent SRH	Consistent FRH	Total	χ^2 (df)	p^a
Marital status	Married	36 (-0.2)	14 (0.3)	53	1.74 (1)	0.322
	Single/widowed/divorced [b]	5 (0.6)	0 (-1.1)	5		
	Total	44	14	58		
Family role	Mother	36 (-0.1)	12 (0.1)	48	0.36 (2)	0.837
	Grandmother	7 (0.1)	2 (-0.1)	9		
	Sister/aunt/stepmother [b]	1 (0.3)	0 (-0.5)	1		
	Total	44	14	58		
Education	No school attendance	40 (-0.2)	14 (0.3)	54	1.37 (1)	0.563
	School attendance [b]	4 (0.6)	0 (-1.0)	4		
	Total	44	14	58		
Literacy	Neither read nor write	42 (-0.1)	14 (0.1)	56	0.66 (1)	0.999
	Only read/read and write [b]	2 (0.4)	0 (-0.7)	2		
	Total	44	14	58		
Religion	Traditional beliefs	42 (0.2)	12 (-0.3)	54	1.57 (1)	0.243
	Muslim/Christian/other [b]	2 (-0.6)	2 (1.1)	4		
	Total	44	14	58		
Livelihood	Pastoralism	43 (0.0)	14 (0.1)	57	0.32 (1)	0.999
	Farming + pastoralism/other [b]	1 (0.3)	0 (-0.5)	1		
	Total	44	14	58		

Note. SR = Standardised residuals. SRH = Stool-related handwashing. FRH = Food-related handwashing.
[a] Bonferonni corrected significance $p < 0.005$.
[b] The remaining categories were merged into one category due to very low frequencies.

References

1. Bender R, Lange S. Adjusting for multiple testing—when and how? J Clin Epidemiol. 2001;54(4):343-9. doi: 10.1016/S0895-4356(00)00314-0.
