# Supplementary material for: Over-Reporting in Handwashing Self-Reports: Potential Explanatory Factors and Alternative Measurements
Source: PLoS One. 2015 Aug 24;10(8):e0136445. doi: 10.1371/journal.pone.0136445 (PMC4547747; doi:10.1371/journal.pone.0136445)
Supplement: S2 File — (PDF) [file pone.0136445.s002.pdf]

S2 File:

Alternative self-report measures to mitigate question sensitivity

Over-reporting in handwashing self-reports: Potential explanatory factors and alternative measurements

Nadja Contzen[1*], Sandra De Pasquale[2], Hans-Joachim Mosler[1]

[1] Environmental and Health Psychology, Department of Environmental Social Sciences, Eawag: Swiss Federal Institute of Aquatic Science and Technology, Duebendorf, Switzerland

[2] Department of Psychology, University of Zurich, Zurich, Switzerland

[*] Corresponding author

E-mail: nadja.contzen@eawag.ch

Introduction

To reduce the effects of question sensitivity, it has been suggested to vary the wording of a question or the context of a question so as to encourage honest, potentially embarrassing responses [1]. A first approach suggests *loading* the wording of a question through presupposing the neglect of a desired behaviour (e.g. instead of asking about washing hands, ask about *not* washing hands) or the engagement in an undesired behaviour [1, 2]. Alternatively, *forgiving wording* could be applied, that is, to introduce a sensitive question with a reasonable excuse for the neglect of a desired behaviour (e.g. there is not enough time or water for handwashing; [1-3]). Similarly, it has been suggested to embed a sensitive question in a *permissive context*, namely to precede the question with a statement that opposes the desired behaviour (e.g. regular handwashing uses up too much water). A *restrictive context* on the other hand, that is, a statement that supports the desired behaviour (e.g. caring mothers wash hands regularly), is expected to increase the effects of question sensitivity [3, 4]. Only a few studies have tested the capacity of these techniques to mitigate socially desirable responding, the majority of which did not successfully prevent socially desirable responding (for an overview, see [1]). Nevertheless, the present study tested these approaches and expected the following:

H12-1: Loaded question wording causes lower self-reported handwashing than unloaded question wording.

H12-2: Forgiving wording with loaded question wording causes lower self-reported handwashing than loaded question wording alone.

H12-3: A permissive context with loaded question wording causes lower self-reported handwashing than loaded question wording alone.

H12-4: A restrictive context with loaded question wording causes higher self-reported handwashing than loaded question wording alone.

Methods

To test alternative self-report measures expected to mitigate socially desirable responding (H12-1–H12-4), an experimental design was applied by using four different questionnaire versions which were randomly assigned to the participants.

Measures

From the four applied questionnaire versions, the first version used only a loaded question wording by asking ‘How often does it happen that you *don’t* wash your hands with soap before handling food or after contact with stool?’ (0 = *almost never/0–1 out of 10 times* to 1 = *almost always/9–10 out of 10 times*). The same item – let us name it item A – was used in the other three versions in combination with an additional item per version. The second version measured forgiving wording by introducing the item A with the sentence ‘Many people don’t have the time to always wash their hands with soap before handling food and after contact with stool’. The third version applied a permissive context by asking the following question ahead of the item A: ‘Do you agree with the following statement: “It does not pay to wash hands with soap before handling food and after contact with stool”?’ (0 = *don’t agree*; 1 = *agree*). A restrictive context was assessed in the fourth version by asking the following question before the item A: ‘Do you agree with the following statement: “Only women who wash hands with soap before handling food and after contact with stool are caring mothers”’. All items were based on previous research [3, 4]. Because these items did not ask for separate key times for handwashing, in order to compare them with the standard self-report items that measured stool- and food-related handwashing separately, the latter were averaged.

Analyses

Whether these alternative self-report measures mitigate the response bias was assessed as follows. To test H12-1, a dependent *t*-test compared loaded question wording to standard self-reported handwashing (averaged stool- and food-related handwashing). An independent *t*-test compared forgiving wording (with loaded question wording) to loaded question wording alone, testing H12-2. An analysis of variance compared a permissive and a restrictive context (both with loaded question wording) to loaded question wording alone (H12-3 and H12-4). All tests applied bootstrapping with 10,000 resamples.

Results and discussion

Our additional research question was as follows: Do alternative self-report measures thought to reduce the influence of socially desirable responding mitigate the response bias? Loaded question wording (M = 0.74, SD = 0.25) did not significantly differ from standard self-reports (M = 0.71, SD = 0.23); t (143) = 1.43, p >. 05, d = 0.12. The same pattern was found for forgiving wording with loaded question wording (M = 0.77, SD = 0.23) compared to

loaded question wording alone (*M* = 0.74, *SD* = 0.25); t (282) = -1.07, p >. 05, d = 0.13. Furthermore, no differences were found between questions applying a permissive (*M* = 0.75, *SD* = 0.27) or a restrictive context (*M* = 0.74; *SD* = 0.27) with loaded question wording or loaded question wording alone (*M* = 0.74, *SD* = 0.25); $F(2, 411) = 0.08$, $p > .05$, $\eta^2 = .00$. That is, H12-1 to H12-4 were all not supported.

In sum, none of the alternative wordings influenced self-reported handwashing. This is in line with previous research on alternative question wording that revealed only limited effects on socially desirable responding (for overviews, see [1, 5]). Moreover, there is some anecdotal evidence from the present study that the negation used in the loaded question wording (which was applied in all alternative wordings) caused some confusion among respondents and interviewers. Overall, the applied alternative question wordings seem to be ineffective in mitigating over-reporting of handwashing. If still applied, the use of negations should be avoided.

References

1. Tourangeau R, Yan T. Sensitive questions in surveys. Psychol Bull. 2007;133(5):859–883. doi: 10.1037/0033-2909.133.5.859.
2. Weisberg HF. The total survey erroer approach: A guide to the new science of survey research. Chigaco, IL: The University of Chicago Press; 2005.
3. Näher A-F, Krumpal I. Asking sensitive questions: The impact of forgiving wording and question context on social desirability bias. Qual Quant. 2012;46(5):1601–1616. doi: 10.1007/s11135-011-9469-2.
4. Tourangeau R, Smith TW. Asking sensitive questions: The impact of data collection mode, question format, and question context. Public Opin Quart. 1996;60(2):275–304. doi: 10.1086/297751. PubMed PMID: 9701143845.
5. Holbrook AL, Krosnick JA. Social desirability bias in voter turnout reports. Public Opin Quart. 2010;74(1):37–67. doi: 10.1093/poq/nfp065.
